# The relationship between social support and professional identity of health professional students from a two-way social support theory perspective: chain mediating effects of achievement motivation and meaning in life

**DOI:** 10.1186/s12909-024-05391-5

**Published:** 2024-04-29

**Authors:** Jian Luo, Xiao-Bo Liu, Qian Yao, Yi Qu, Jin Yang, Ke Lin, Shi-Rong Pan, Tian-Yi Wang, Yun Dai, Huan-Yu Chen, Jian-Min Chen, Zheng Yang

**Affiliations:** 1 School of Nursing, Chengdu Medical College, Chengdu, 610500 China; 2 School of Acupuncture and Tuina, Chengdu University of Chinese Medicine, Chengdu, 611137 China; 3 Security Department of Chengdu Medical College, Chengdu, 610500 China; 4 School of Clinical Medicine, Chengdu Medical College, Chengdu, 610500 China; 5 School of Basic Medicine, Chengdu Medical College, Chengdu, 610500 China

**Keywords:** Two-way social support, Achievement motivation, Meaning in life, Professional identity, Mediating effect

## Abstract

**Background:**

Studies has suggested that receiving social support improves the professional identity of health professional students. According to the two-way social support theory, social support includes receiving social support and giving social support. However, the effect of the two-way social support on health professional students’ professional identity has not been clarified yet.

**Methods:**

To explore the mechanism of how two-way social support affects health professional students’ professional identity, an observational, cross-sectional study was conducted among a convenience and cluster sample of 1449 health professional students from two medical schools in western China. Measures included a short version of the two-way social support scale, a health professional students’ professional identity questionnaire, an achievement motivation scale, and a meaning in life scale. (Data were analyzed by use of SPSS26.0 software, Amos 28.0 software, and PROCESSv4.0 plug-in.).

**Results:**

Receiving social support, giving social support, achievement motivation, meaning in life, and professional identity were positively correlated with each other. Receiving and giving social support not only directly predicted health professional students’ professional identity, but also indirectly predicted health professional students’ professional identity through the mediating roles of achievement motivation and meaning in life, and the chain mediating roles of achievement motivation and meaning in life, respectively. The effectiveness of predicting health professional students’ professional identity varied among different types of two-way social support, which could be depicted as two-way social support > mainly giving social support > mainly receiving social support > low two-way social support.

**Conclusion:**

In the medical education, the awareness and ability of health professional students to receive and give social support should be strengthened. More attention should be drawn on the chain mediating effect of achievement motivation and meaning in life between two-way social support and professional identity. The current results shed new light on exploring effective ways of improving health professional students’ professional identity, which suggested that more attention should be paid to the positive effects of mainly giving social support and two-way social support rather than only on the effects of receiving social support.

## Introduction

### Professional identity of health professional students

Professional identity is believed to improve self-confidence and resilience in health professionals. Health professional students are future members of health care services. Therefore, professional identity formation (PIF) of health professional students is of great importance to public health services and their own future careers. Professional identity formation of health professional students is a dynamic process based on their beliefs and values [1], professional education [2] and social interactions with others at workplaces [3–5]. Professional identity formation (PIF) is initiated before medical education [6]. Community practice theory furnishes a theoretical framework for professional identity formation [7]. Medical students actively engage with and assimilate into the clinical and teaching environments through participation in medical communities (involving communication, interaction, and mutual support among healthcare teams, research institutions, and patient groups), the pursuit of shared goals (common practice objectives, career goals, and learning goals), and resource sharing. This process allows them to discover a sense of belonging and accomplishment, facilitating the transition from basic learning and work cognition to professional identity [8, 9]. The Personality Ring Theory (RToP) also asserts that community participation and interpersonal interaction play crucial roles in promoting the formation of professional identity [10].

The professional identity of health professional students is also influenced by a variety of demographic, social, and psychological factors [5, 11]. Professional identity has been proved to reduce academic burnout [12], enhance readiness for hospital practice [13], and alleviate role stress among nursing students during clinical placements [14]. Improving the professional identity of health professional students is of great importance to the development of the health system [11]. Events, such as the new medical reform policy, the running of a standardized training system, tense doctor-patient relationships, and negative remarks from the public, have presented intense pressures on both health professionals and health professional students, which would eventually impact health professional students’ professional identity [5]. The formation and maintenance of health professional students’ professional identity has raised increasing attention in recent years.

### The influence of social support on health professional students’ professional identity

To develop effective ways of improving health professional students’ professional identity, researchers have tried to seek theories that demonstrate the underlying mechanisms of professional identity formation. Social support was believed to be a key factor in this process. The direct effects model of social support shows that social support networks can directly lead to positive psychological states, including a sense of purpose, belonging, security, and self-identity [15, 16]. Based on Maslow’s hierarchy of needs theory [17], it can be found that receiving or sensing social support from classmates, teachers, or health professionals can help individuals find a sense of belonging in the learning or working environment. Giving professional-related social support to others is more likely to enable individuals to realize their self-worth, thus satisfying their needs at a higher level and forming professional identity. In view of functional aspects, social support theory suggested that social support contained four main characters-emotional, instrumental, informational, and appraisal [18]. The existing four-factor [19] and five-factor [20] models of social support represent modifications of the original model [21]. Numerous assessment tools of social support, such as perceived social support and social support questionnaires, were developed based on this theory [22, 23]. In 1987, Maton [24] first proposed the two-way social support theory. Maton suggested that social support included not only receiving social support but also giving social support and that individuals of higher levels of two-way social support were more likely to maintain positive psychological conditions than those of one-way social support (mainly receiving or giving social support). Compared to the direct effects model of social support and social support theories that focus solely on receiving social support, the two-way social support theory suggests that giving social support seems to better promote medical students’ community engagement and interpersonal interactions, thereby enhancing their sense of professional identity.

During the COVID-19 pandemic, although health professional students reported high levels of receiving social support in terms of support, they felt isolated and lacked social support [25]. This suggests that simply receiving social support may not meet the health, psychological, and social development needs of health professional students. Providing social support is a factor that researchers should consider. The importance of giving social support among health professional students has been preliminarily explored. The volunteer service motivation of Medical students centered on values has been shown to regulate their levels of happiness [26]. Medical students actively involved in community volunteer services showed significant improvement in cultivating professional skills, mastery of knowledge, and development of professional interests compared to non-volunteer students [27]. With an increase in volunteer service time, medical students reported an improvement in their quality of life, and perceived stress levels significantly decreased [28].

Previous studies showed that receiving and giving social support could positively predict professional identity, which has been verified among medical workers [29, 30], health professional students [31, 32], and teachers [33]. However, how to enhance professional identity through social support still needs to be further explored. The majority of relative studies mainly concentrated on receiving social support. Still some researches explored the role of both receiving and giving support in the formation of professional identity. As was proved that social support was characterized of reciprocity and equilibrium [34, 35], participants who performed giving social support while receiving social support had less psychological burdens [36]. Other studies found that health professional students who had taken part in voluntary activities during COVID-19 had higher levels of professional identity [5, 37]. Tong et al. [38] found that nurses who had cared for patients infected with COVID-19 were 17.95 (95% CI: 2.38-135.39, *P* = 0.005) times more likely to have stronger professional identity compared to nurses who had not. Therefore, both giving and receiving social support could possibly facilitate an individual’s professional identity. Based on the results of relevant studies and the two-way social support theory, we hypothesized that receiving and giving support were both positively associated with professional identity (Referred as H1 in this article).

### The mediating effect of achievement motivation

Self-determination theory posits that motivation can be categorized into two types: autonomous and controlled motivation [39]. The autonomous motivation of medical students can enhance professional identity and facilitate the formation of professional identity [6]. Achievement motivation falls under autonomous motivation, achievement motivation was defined as the internal motivation of an individual to achieve success in completing tasks, including the pursuit of success and avoiding failure. The motivation of an individual to pursue success was determined by the expectation of success, the evaluated possibility of success, and the incentive of success. Pursuit of success was a promoting factor of achieving one’s goals, while avoiding failure would prevent one’s goals from being realized [40, 41]. Achievement motivation is considered a positive factor in promoting student growth, as it can reduce academic fatigue [42], enhance students’ proactive learning abilities, and boost academic confidence [43]. Receiving social support provided individuals with financial and spiritual support which was needed for their success [44]. Therefore, receiving social support could help a person make higher estimation on the possibility of success and thus enhance their motivation to pursue success. Studies found that receiving social support was positively correlated with achievement motivation among college students and adolescents [45–47]. According to extended construction theory [48], giving social support could help individuals explore potential social resources (such as interpersonal resources), which was also of great significance for achieving successes. In addition, compared to receiving social support, improving the ability to give social support helps to stimulate internal motivations (such as confidence, self-efficacy, etc.). Therefore, giving social support can also promote individuals’ estimation on the possibility of success and improve their motivation to pursue success by supporting them both socially and psychologically. A study on college students showed that achievement motivation can increase the level of professional identity of college students majoring in preschool education [49], which was consistent with the results of Ge [50] on general practitioners. Above all, we hypothesized that achievement motivation might have mediating effects between receiving and giving social support and professional identity (Referred as H2 in this article).

### The mediating effect of meaning in life

Meaning in life(MIL) refers to the realization of the importance of life and the sense of aims and goals in life-based on one’s understanding of self and relationship with the external environment [51]. Based on the related concepts of “meaning of life” [51–53], Chinese scholars concluded that MIL is a process in which individuals perceive the purpose and meaning of their existence, and make continuous efforts and continuous thinking about the goals, values, and meanings of life [54]. The achievement of meaning in life yields various benefits, including a reduction in the risk of depression among vocational medical school students [55], an increase in life satisfaction of postgraduate medical students [56], and an enhancement of overall health levels among health professional students [57]. It bears significant importance for the holistic development of physical, psychological, and social well-being. According to self-determination theory, individuals develop personality perfection and psychological maturity during integrating their goals with motivations, which facilitates the sense of meaning in life. In this process, social support plays an indispensable role by promoting the internalization of external motivations [58]. In addition, the conceptual model of meaning in life proposes that giving social support plays a positive role in the construct of meaning in life [59]. Numerous studies further confirmed the positive correlation between social support and meaning in life [60–62]. Another study on clinical medical freshmen found that meaning in life was positively correlated with professional identity [63]. Based on these evidences, we hypothesized that meaning in life had a mediating effect between receiving and giving social support and professional identity (Referred as H3 in this article).

### The chain mediating effect of achievement motivation and meaning in life

Studies on health professional students showed that the motivation to pursue success and avoid failure was significantly related to meaning in life [64]. The same conclusion was drawn in another study on Finnish college students [65]. Some studies also confirmed that achievement motivation positively predicted meaning in life in the general population. It seemed that achievement motivation and meaning in life not only had mediating effects between social support and professional identity respectively, but also had chain mediating effects in this process. Based on these findings, we hypothesized that achievement motivation and meaning in life had chain mediating effects between social support and professional identity (Referred as H4 in this article). In addition, the theory of two-way social support suggests that two-way social support could improve positive emotions more than one-way social support. Therefore, we hypothesized that two-way social support was more efficient than one-way social support in predicting professional identity both directly and through the chain mediating effect of achievement motivation and meaning in life (Referred as H5 in this article).

**Fig. 1:**
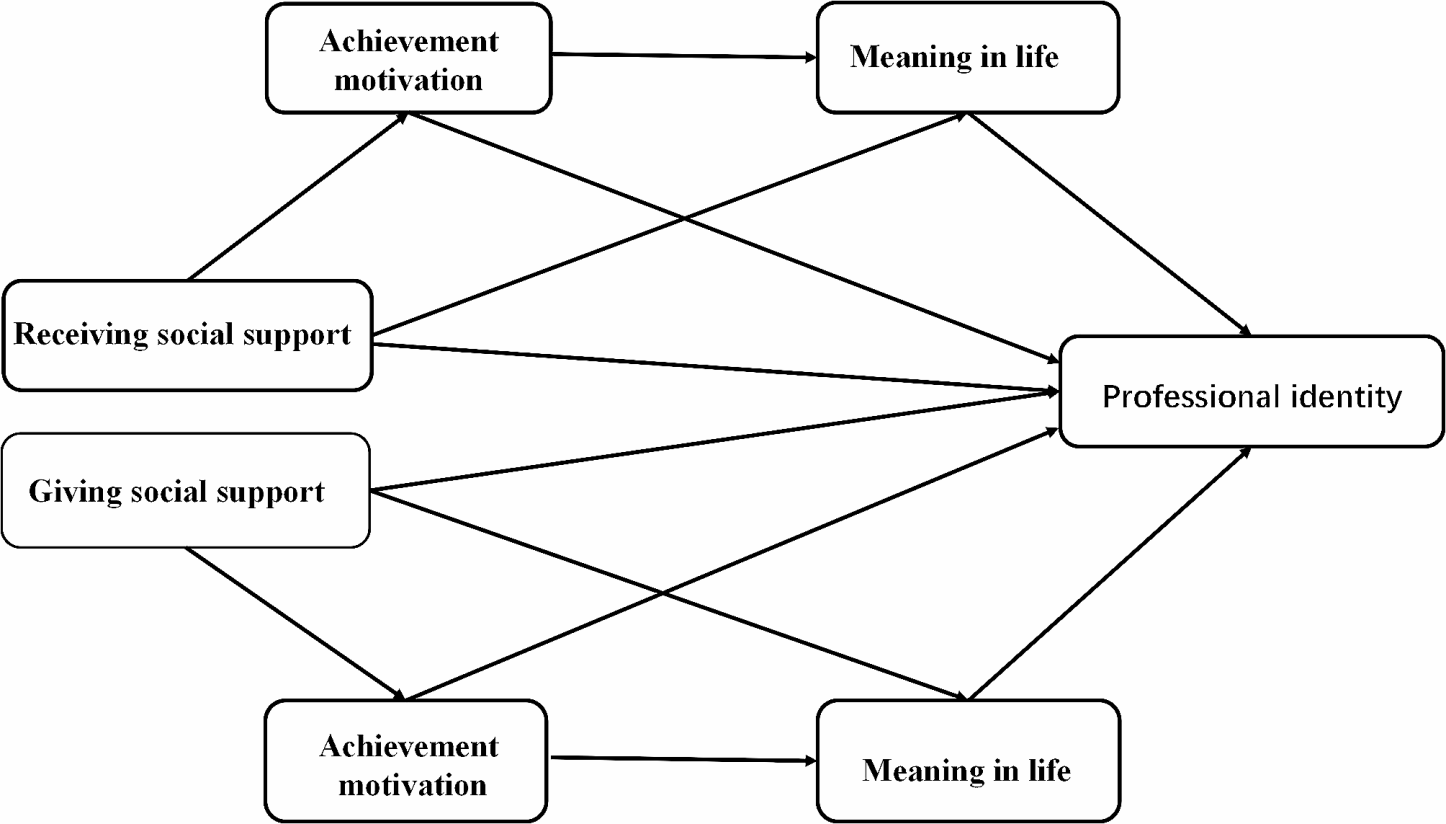
The theoretical model of the chain intermediary relationship between two-way social support and professional identity

In summary, according to hypothesis H1-4, we constructed a chain mediation model for the relationship between two-way social support and professional identity through the chain mediating effect of achievement motivation and meaning in life (as shown in Fig. 1).

## Materials and methods

### Procedure and participants

Undergraduates majoring in medicine (excluding health professional students during internship) from a medical college and a university of Traditional Chinese Medicine in western China were recruited through convenience sampling in this study. Each school was represented by a research team consisting of two individuals. Questionnaires were distributed on the Wenjuanxing platform (https://www.wjx.cn/) from November 1, 2022, to February 1, 2023. To control the quality of the questionnaires returned, researchers conducted a pre-survey with 15 participants before finalizing the questionnaire. The shortest completion time for the questionnaire under careful investigation was determined to be 75 s. Based on the distribution of students in each major, researchers proportionally selected the corresponding number of participants from each major and grade. Participants were introduced by class monitors or grade counselors. Researchers had contacted the participants through QQ or WeChat before the survey. Informed consent was obtained from every participant, and instruction (All items were mandatory, and the completion time had to be greater than 75 s for the questionnaire to be considered valid.) of the survey was explained to them. Upon submission of the questionnaire, participants were provided a small incentive distribution link through the Wenjuanxing platform. A total of 1449 questionnaires were collected, of which 47 were deemed invalid (11 due to a patterned response and 36 with a completion time below 75 s). The study obtained 1402 valid samples. Before commencing the research, the research team submitted an ethical application to the Ethics Committee of Chengdu Medical College, and the study received approval from the committee.

### Measures and instruments

#### General information questionnaire

The members of the research group jointly formulated the general information questionnaire, including age, gender, school attended, major studied, etc.

### Assessment of professional identity

A questionnaire of professional identity for health professional students compiled by Zhang et al. [66] was applied. The questionnaire included three items: (1) Strong interests and curiosities in the medical profession or a particular field of medicine; (2) Being proud of one’s future career in medicine; (3) Being willing to choose medicine as one’s major had been given a second chance. The questionnaire used a 5-point Likert scale, ranging from “strongly disagree” to “strongly agree”. The higher the score, the greater the professional identity. The questionnaire has been applied to clinical medical undergraduates [42], and its Cronbach’s α value was 0.78. The Cronbach’s α value in this study was 0.72. It was a short and reliable professional identity assessment tool.

### Assessment of social support

Existing social support assessment scales, such as the Social Support Questionnaire and the Perceived Social Support Questionnaire, are widely used. However, they tend to assess emotional or structural social support, and the bidirectionality of social support has not been adequately considered [21]. The Simplified 2-way Social Support Scale was developed by Obst et al. [21, 67]. The Simplified Bidirectional Social Support Scale was initially translated into Chinese and validated by Lin and colleagues [68] among individuals under 30 years old, specifically targeting primary school teachers. The questionnaire demonstrated good reliability, with Cronbach’s α coefficients of 0.94 for the overall scale, 0.93 for the giving social support dimension, and 0.91 for the receiving social support dimension. Confirmatory factor analysis results (χ²/df = 3.22, RMSEA = 0.08, CFI = 0.94, TLI = 0.93, SRMR = 0.04) indicated that the scale also exhibited satisfactory validity among the younger population. Subsequently, Cui et al. [69] conducted a second translation, incorporating bidirectional translation, expert consultation, cultural adaptation, and pre-investigation, followed by testing among community-dwelling older adults. The fit indices from confirmatory factor analysis were satisfactory (χ²/df = 2.421, RMR = 0.029, GFI = 0.952, CFI = 0.989, IFI = 0.989, TLI = 0.985, RMSEA = 0.061). Additionally, Cronbach’s α coefficients for each dimension were greater than 0.91, and test-retest reliability exceeded 0.74, with a split-half reliability exceeding 0.9. The scale consists of four dimensions: receiving emotional support, giving emotional support, receiving tool support, and giving tool support, with 12 entries using a Likert 5-level scoring method with a total score in the range of 12–60 points. The higher the score, the higher the two-way social support level. The total Cronbach’s α value was 0.955. The Cronbach’s α value of social support (including emotional support and tool support) was 0.887. The Cronbach’s α value of social support (including emotional support and tool support) was 0.909.

### Assessment of achievement motivation

The short version of the Achievement Motivation Scale (AMS) was developed by Gjesme and Nygard and was revised by Ye et al. in 1988 [70]. The scale was used to measure the motive to achieve success (Ms) and the motive to avoid failure (Mf). Later, Tang et al. [71]developed a Short Form of Achievement Motive Scale (AMS-SF) based on the short version. The AMS-SF consists of two dimensions: motive for success and motive for avoiding failure, with 6 entries using a Likert 4-level scoring method. The Likert 4 scale is scored with 4 points for being “completely correct”, 3 points for being “mostly correct”, 2 points for being “somewhat correct” and 1 point for being “completely wrong”. The higher the score, the stronger the achievement motivation. The scale has been widely utilized among Chinese health profession students and clinical professionals, with Cronbach’α coefficients for the scale exceeding 0.85 in these populations. The Cronbach’α value was 0.913.

### Assessment of the meaning in life

Purpose in Life (PIL) Scale [72] is not only the most commonly used tool for measuring the meaning in life in China but also the most important scale for the meaning in life of health professional students. Schulenberg et al. developed the Purpose in Life Test-Short Form (PIL-SF) Scale by selecting 4 entries from the PIL [73]. The scale utilizes a 7-level scoring method, with the total score in the range of 4–28 points. The higher the score, the stronger the sense of goals and meaning in life. Xiao Rong et al. [74] converted the scale into Chinese and tested its reliability and validity among college students. The Cronbach’s α value was determined as 0.87 and was retested as 0.786. In this study, the Cronbach’s α value was 0.862.

### Statistical analyses

Excel was used to collect and sort out the data. The data were analyzed by SPSS 26.0 software(IBM SPSS Statistics for Windows, Version 26.0. Armonk, NY; IBM Corp.), PROCESS plug-ins (SPSS PROCESS macro, version 3.4, developed by Preacher and Hayes), and Amos 28.0 (Amos Development Corporation). The analysis was performed as follows: (1) The common method deviation test was carried out for data analysis. (2) Descriptive statistical methods were conducted to analyze the data about the general information of the participants by using SPSS26.0. Pearson bivariate correlation was used to evaluate the relationship among main variables, including two-way social support, achievement motivation, meaning in life, and professional identity. (3) Based on the research hypotheses, three structural equation models were constructed using Amos 28.0. Model 1: Examining the mediating roles of achievement motivation and meaning in life in the relationship between receiving social support and professional identity. Model 2: Examining the mediating roles of achievement motivation and meaning in life in the relationship between giving social support and professional identity. Model 3: Examining the separate mediating roles of achievement motivation and meaning in life in the relationship between both receiving and giving social support and professional identity. Model fit was assessed using various indices, including the Comparative Fit Index (CFI), Normal Fit Index (NFI), Tucker-Lewis Index (TLI), Root Mean Square Error of Approximation (RMSEA), Goodness-of-Fit Index (GFI), Chi-square Minimum/Degree of Freedom (χ²/df), and Standardized Root Mean Square Residual (SRMR). Acceptable model fit was indicated by RMSEA < 0.08, CFI, GFI, NFI, TLI > 0.9, χ²/df < 5, and SRMR < 0.08 [75, 76]. (4) The bias-corrected percentile Bootstrap method in Amos was employed to test the chain-mediated effects of achievement motivation and meaning in life. (5) Evaluate how different types of social support predict professional identity via direct effects or indirect effects of achievement motivation and meaning in life if the chain mediating effect had been verified. Participants were ranked in order of their social support scores by using SPSS26.0 and divided into the following 2 groups: high-score group and low-scoring group. The top-27%-scoring participants (score > 21.9) were clustered into a high-score group and the rest were in a low-score group [77]. The high-score group was further divided into 4 subgroups according to Maton [24] ‘s classification method. Model 6 of the PROCESS plug-in with dummy variables representing the categorical variables was used to test the direct and indirect effects of different types of two-way social support on health professional students’ professional identity [78]. A P-value of < 0.05 was considered statistically significant. The chain mediating effects (relatively) were considered significant if 0 was not included in the Bootstrap confidence intervals.

## Results

### Socio-demographic characteristics of participants

A total of 669 health professional students from the University of Traditional Chinese Medicine and 780 health professional students from the medical school were included in this study. 1402 participants provided usable data. There were 396 male students (28.2%) and 1006 female students (71.8%), aged between 17 and 25 years old. Other socio-demographic information, such as grade, place of residence, and major, is shown in Table 1.

**Table 1:** General statistics of health professional students

Projects	Category	Proportion(%)	Projects	Category	Proportion(%)
Age	17 ~ 18	14.7	Place of residence	Countryside	34.7
	19	19.2		Town	26.6
	20	30.4		City	38.7
	21	23.2	Major	Nursing	30.8
	22 ~ 25	12.5		Clinical medicine	18.0
Sex	male	28.2		Traditional Chinese medicine	9.8
	female	71.8		Medical imaging	9.0
Grade	Freshman year	24.8		Acupuncture and massage	8.4
	Sophomore year	29.2		Medical laboratory science	5.3
	Junior year	38.2		Anesthesia medicine	4.1
	Senior year	7.8		The other eight medical majors	14.6

### Assessment of common method variance

The Harman single-factor method was used to test the common method bias. The results showed that the latent roots of 5 factors were greater than 1. The explanation rate of the first factor for the variables was 26.49%, which was lower than the standard value of 40%. Therefore, common method bias was controlled in this study.

Descriptive statistical analysis and correlation analysis of each variable.

The scores of the relevant variables and the results of the correlation analysis are shown in Table 2. Receiving social support, giving social support, achievement motivation, and meaning in life were positively correlated to professional identity (*p* < 0.01). Receiving social support, giving social support, and achievement motivation were positively correlated (*p* < 0.01), and receiving social support was positively correlated with giving social support (*p* < 0.01). The correlation coefficients ranged from 0.139 to 0.849. Further mediation effect tests could be conducted.

**Table 2:** Statistical analysis and correlation analysis of research variables

	1	2	3	4	5
Receiving social support	1				
Giving social support	0.849**	1			
Achievement motivation	0.150**	0.139**	1		
Meaning in life	0.445**	0.463**	0.354**	1	
Professional identity	0.500**	0.538**	0.229**	0.509**	1
Mean	3.872	3.932	-0.570	5.361	3.714
SD	0.790	0.716	5.226	1.100	0.841

*Note* ** *P* < 0.01

**Fig. 2:**
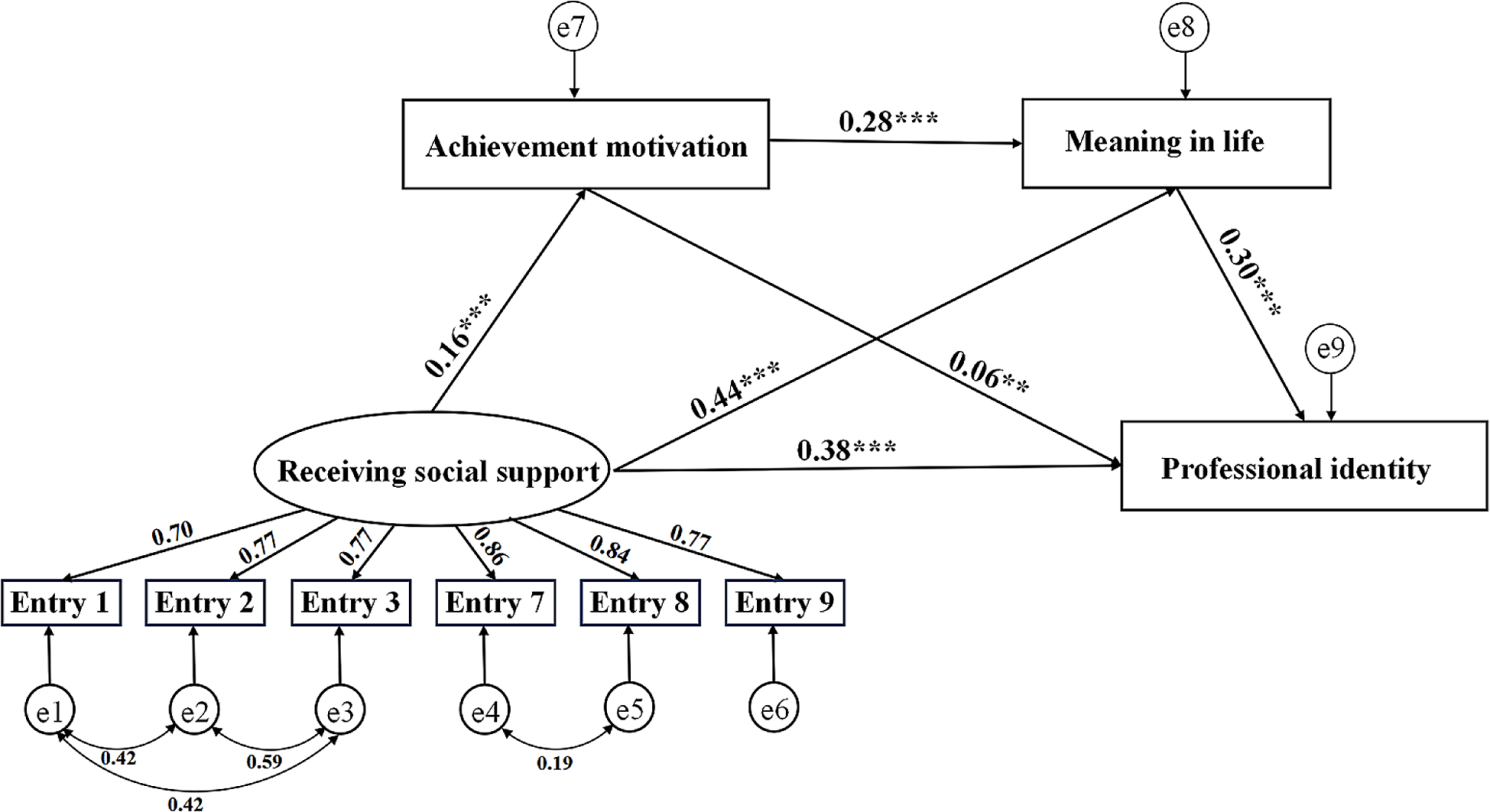
The structural equation model of receiving social support, achievement motivation, meaning in life, and professional identity. *Note* ** *P* < 0.01; ***: *P* < 0.001

### Construction of structural equation model

**Fig. 3:**
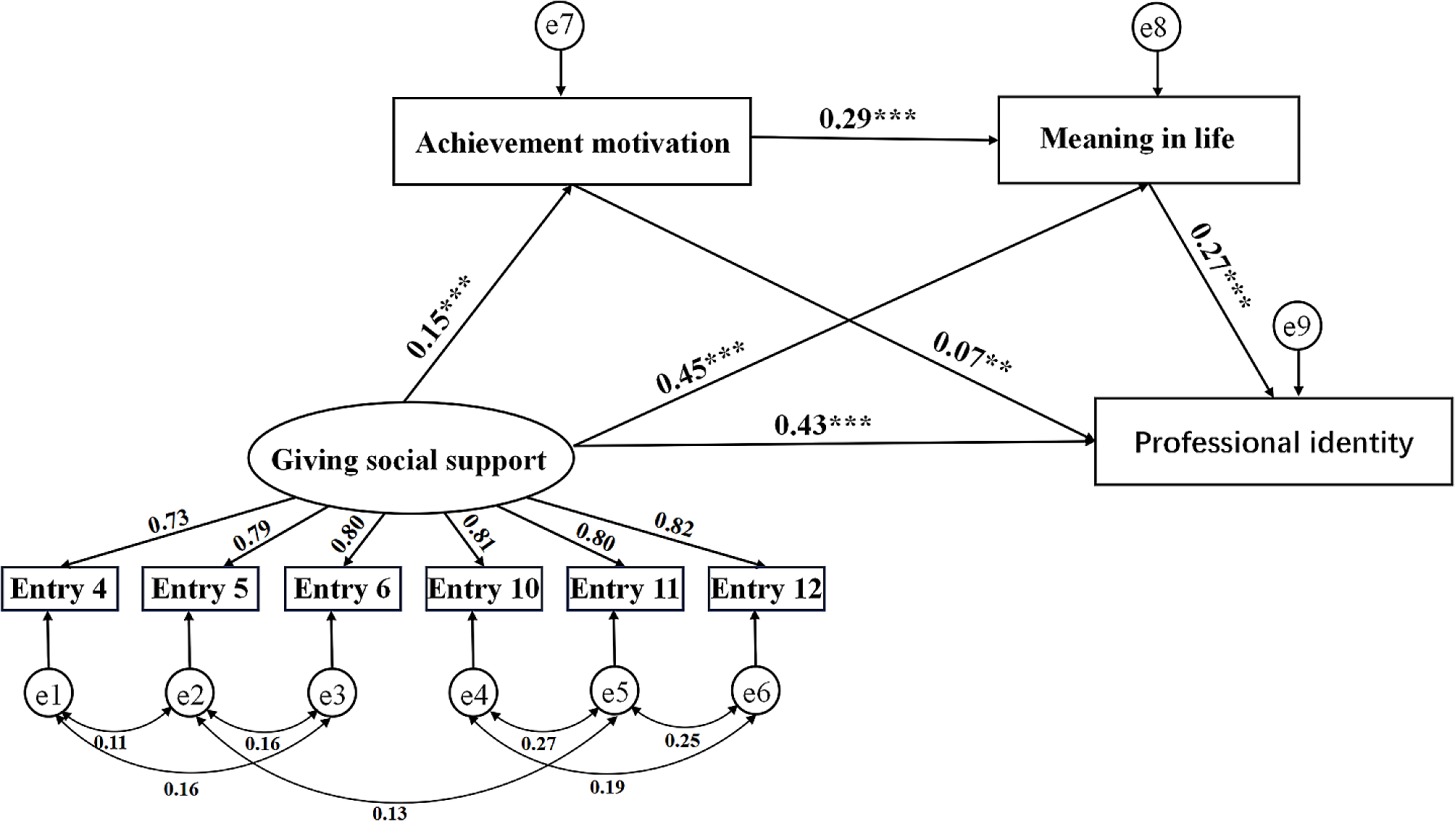
The structural equation model of receiving social support, achievement motivation, meaning in life, and professional identity. *Note* ** *P* < 0.01; ***: *P* < 0.001

**Fig. 4:**
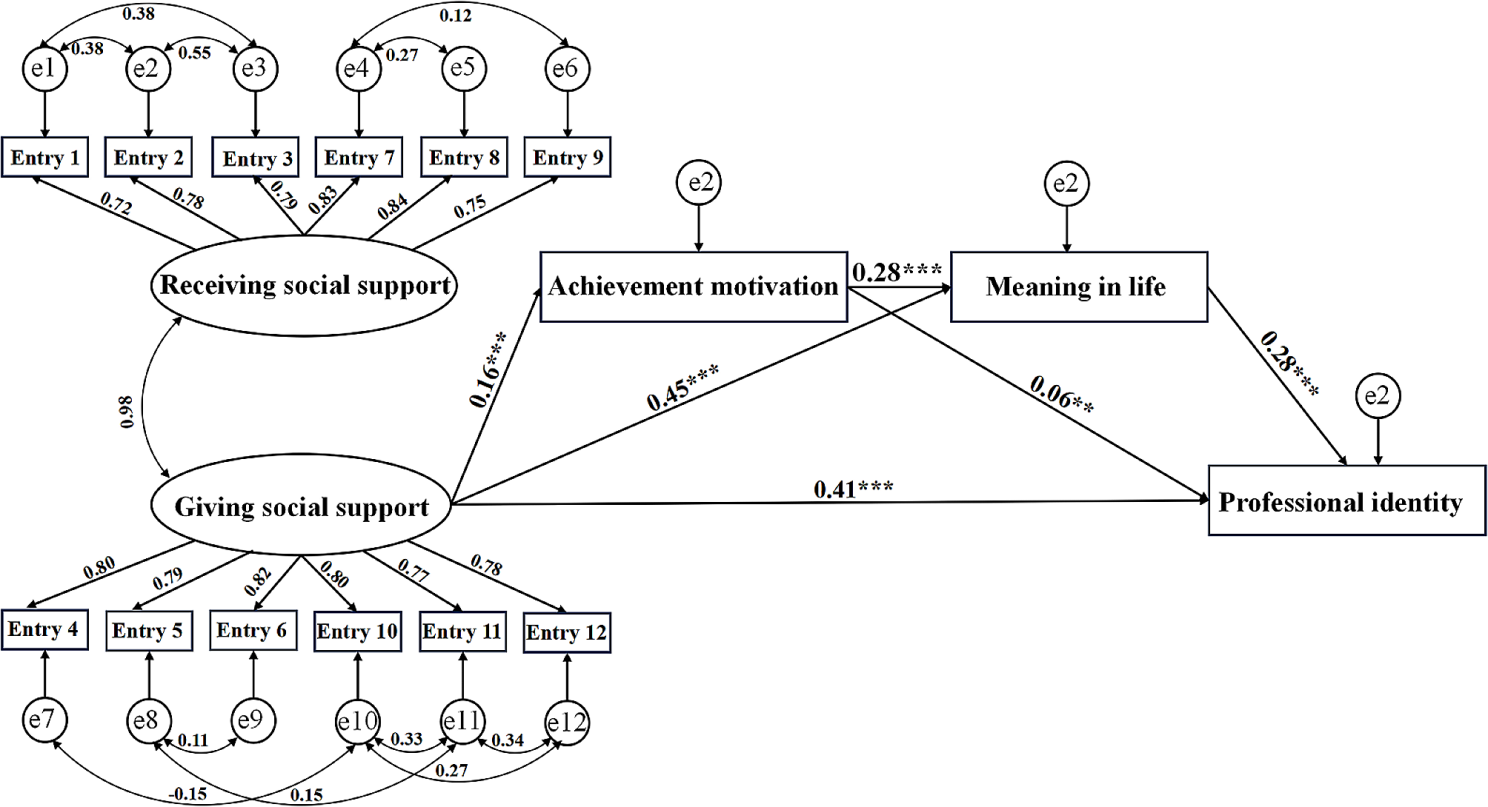
The structural equation model of receiving social support, achievement motivation, meaning in life, and professional identity. *Note* ** *P* < 0.01; ***: *P* < 0.001

Using AMOS 28.0, three structural equation models were constructed: Model 1 (predicting professional identity through the mediation of achievement motivation and meaning in life by acceptance of social support), Model 2 (predicting professional identity through the mediation of achievement motivation and meaning in life by provision of social support), and Model 3 (predicting professional identity through the mediation of achievement motivation and meaning in life by both acceptance and provision of social support). Preliminary construction of Models 1, 2, and 3 revealed that the fit indices (χ²/df) for each original model were greater than 5. Therefore, based on modification indices (MI), the models were adjusted. The adjusted Model 1 had fit indices (χ²/df = 2.257, RMSEA = 0.03, SRMR = 0.01, CFI = 0.99, TLI = 0.99, GFI = 0.99), the adjusted Model 2 had fit indices (χ²/df = 2.68, RMSEA = 0.04, SRMR = 0.01, CFI = 0.99, TLI = 0.99, GFI = 0.99), and the adjusted Model 3 had fit indices (χ²/df = 4.91, RMSEA = 0.05, SRMR = 0.02, CFI = 0.98, TLI = 0.97, GFI = 0.97) within acceptable ranges. The structural equation model reports standardized coefficients and their significance. The results for Model 1 are shown in Fig. 2. Receiving social support positively predicted professional identity (*r* = 0.38, *p* < 0.001). Receiving social support also positively predicted achievement motivation (*r* = 0.16, *p* < 0.001) and meaning in life (*r* = 0.44, *p* < 0.001). Achievement motivation positively predicted meaning in life (*r* = 0.28, *p* < 0.001) and professional identity (*r* = 0.06, *p* < 0.01). Meaning in life positively predicted professional identity (*r* = 0.30, *p* < 0.001). The results for Model 2 are shown in Fig. 3. Giving social support positively predicted professional identity(*r* = 0.43, *p* < 0.001). Giving social support also positively predicted achievement motivation (*r* = 0.15, *p* < 0.001), and meaning in life (*r* = 0.45, *p* < 0.001). Achievement motivation positively predicted meaning in life (*r* = 0.28, *p* < 0.001) and professional identity (*r* = 0.07, *p* < 0.01). Meaning in life positively predicted professional identity (*r* = 0.27, *p* < 0.001). The results of Model 3 are presented in Fig. 4. In this model, giving social support positively predicts achievement motivation (*r* = 0.16, *p* < 0.001), meaning in life (*r* = 0.45, *p* < 0.001), and professional identity (*r* = 0.41, *p* < 0.001). Achievement motivation positively predicts meaning in life (*r* = 0.28, *p* < 0.001) and professional identity (*r* = 0.06, *p* < 0.01). Meaning in life positively predicts professional identity (*r* = 0.28, *p* < 0.001). However, the predictive effects of receiving social support on achievement motivation, meaning in life, and professional identity are not significant.

### Mediation effects test

The effect of social support on professional identity mediated by achievement motivation and meaning in life was analyzed by bias-corrected percentile Bootstrap method with 5000 repeated samples [79]. The mediating effect was considered to be significant when the 95% confidence interval of the indirect effect value did not include 0. Results of Model 1 showed that the total effect of receiving social support on professional identity was 0.696 (95% CI [0.614,0.775]) with a direct effect of (*r* = 0.492, 95% CI [0.391, 0.590]), accounting for 70.8% of the total effect. Achievement motivation played an independent mediating role between receiving social support and professional identity, with a mediating effect accounting for 1.8% of the total effect. Meaning in life independently mediated the relationship between receiving social support and professional identity (*r* = 0.173, 95% CI [0.127, 0.227]), with a mediating effect of 24.8% of the total effect. Achievement motivation and meaning in life played a chain mediating role between receiving social support and professional identity (*r* = 0.018, 95% CI [0.011, 0.029]), with a mediating effect of 2.6% of the total effect. Differential testing of the mediating effects showed that the indirect predictive effect of receiving social support on professional identity through meaning in life in this model was significantly greater than the other two mediating paths (*p* < 0.001), as detailed in Table 3. Results of Model 2 showed that the total predictive effect of giving social support on professional identity was 0.705 (95% CI [0.636,0.775]), with a direct effect of *r* = 0.527 (95% CI [0.438, 0.619]), accounting for 74.8% of the total effect. Achievement motivation plays an independent mediating role between giving social support and professional identity (*r* = 0.013, 95% CI [0.003, 0.026]), with a mediating effect accounting for 1.8% of the total effect. Meaning in life independently mediates the relationship between giving social support and professional identity (*r* = 0.151, 95% CI [0.107, 0.204]), with a mediating effect of 21.4% of the total effect. Achievement motivation and meaning in life play a chain mediating role between giving social support and professional identity (*r* = 0.014, 95% CI [0.008, 0.024]), with a mediating effect of 2.0% of the total effect. Differential testing of the mediating effects shows that the indirect predictive effect of giving social support on professional identity through meaning in life in this model is significantly greater than the other two mediating paths (*p* < 0.001), as detailed in Table 3. Model 3 results show that the total predictive effect of giving social support on professional identity is 0.721 (95% CI [0.652, 0.790]), with a direct effect of *r* = 0.527 (95% CI [0.437, 0.617]), accounting for 73.2% of the total effect. Achievement motivation plays an independent mediating role between giving social support and professional identity (*r* = 0.013, 95% CI [0.002, 0.027]), with a mediating effect accounting for 1.8% of the total effect. Meaning in life independently mediates the relationship between giving social support and professional identity (*r* = 0.164, 95% CI [0.118, 0.220]), with a mediating effect of 22.7% of the total effect. Achievement motivation and meaning in life play a chain mediating role between giving social support and professional identity (*r* = 0.017, 95% CI [0.010, 0.027]), with a mediating effect of 2.3% of the total effect. Differential testing of the mediating effects shows that the indirect predictive effect of giving social support on professional identity through meaning in life in this model is significantly greater than the other two mediating paths (*p* < 0.001), as detailed in Table 3. Therefore, when both receiving social support and giving social support exist independently, research hypotheses H1-4 are all established. However, when receiving social support and giving social support coexist, achievement motivation and meaning in life only mediate the relationship between giving social support and professional identity, and hypotheses H1-4 are not fully established.

**Table 3:** Mediating effects test using bootstrap analysis

Analytical perspective	Paths	Standardized effect size	Boot LLCI	Boot ULCI	*P* value	Percentage to total effect
Model1: RSS→PI	Dir RSS→PI	0.492	0.391	0.590	< 0.001	70.8%
	Ind1 RSS→AM→PI	0.013	0.002	0.026	0.028	1.8%
	Ind2 RSS→MiL→PI	0.173	0.127	0.227	< 0.001	24.8%
	Ind3 RSS→AM→MiL→PI	0.018	0.011	0.029	< 0.001	2.6%
	Taotal	0.696	0.614	0.775	< 0.001	1
	Difference1(Ind1-Ind2)	-0.160	-0.217	-0.111	< 0.001	/
	Difference2(Ind1-Ind3)	-0.005	-0.022	0.007	0.384	/
	Difference3(Ind2-Ind3)	0.155	0.111	0.207	< 0.001	/
Model 2: GSS→PI	Dir GSS→PI	0.527	0.438	0.619	< 0.001	74.8%
	Ind1 GSS→AM→PI	0.013	0.003	0.026	0.010	1.8%
	Ind2 GSS→MiL→PI	0.151	0.107	0.204	< 0.001	21.4%
	Ind3 GSS→AM→MiL→PI	0.014	0.008	0.024	< 0.001	2.0%
	Total	0.705	0.636	0.775	< 0.001	1
	Difference1(Ind1-Ind2)	-0.138	-0.193	-0.091	< 0.001	/
	Difference2(Ind1-Ind3)	-0.002	-0.016	0.010	0.740	/
	Difference3(Ind2-Ind3)	0.136	0.095	0.187	< 0.001	/
Model 3: (RSS and GSS)→PI	Dir GSS→PI	0.527	0.437	0.617	< 0.001	73.2%
	Ind1 GSS→AM→PI	0.013	0.002	0.027	0.023	1.8%
	Ind2 GSS→MiL→PI	0.164	0.118	0.220	< 0.001	22.7%
	Ind3 GSS→AM→MiL→PI	0.017	0.010	0.027	< 0.001	2.3%
	Total	0.721	0.652	0.790	< 0.001	1
	Difference1(Ind1-Ind2)	-0.151	-0.208	-0.102	< 0.001	/
	Difference2(Ind1-Ind3)	-0.004	-0.019	0.009	0.546	/
	Difference3(Ind2-Ind3)	0.147	0.103	0.201	< 0.001	/

*Note* Dir: Direct effecte; Ind: Indirect effecte; RSS: Receiving social support; GSS: Giving social support; AM: Achievement motivation; MiL: Meaning in life; PI: Professional identity

### Test of predictive efficacy of different bidirectional social support types

To investigate the distinct effects of different bidirectional social support types on professional identity, we divide the participants into 4 subgroups according to their two-way social support scores. It turned out that 417 people (29.7%) belonged to high two-way social support group, 275 people (19.6%) belonged to mainly giving social support group, 285 people (20.3%)belonged to mainly receiving social support group, and 425 people (30.3%) belonged to low two-way social support group. The result was coincident with Maton’s study (33:17:17:33) [24]. The Bootstrap method was used to analyze the direct and mediating effects of high two-way social support, mainly receiving social support and mainly giving social support on professional identity, taken low two-way social support group as a comparison. The results showed a significant overall total effect (R^2^ = 0.1686, F = 94.554, *p* < 0.001) and an overall direct effect (R^2^ = 0.0471, F = 31.695, *p* < 0.001) was significant. The relative effects are shown in Table 4. Compared with the low two-way social support group, the relative mediating effects of high two-way social support and mainly receiving social support on professional identity through achievement motivation were significant (0 not included in the Boot method confidence intervals). This was consistent with the results on the relative mediating effects in the chain mediating regulation of distinct two-way social supports on professional identity through achievement motivation and meaning in life. The relative total and direct effects of distinct two-way social supports on professional identity were significant, and the relative mediating effect of distinct two-way social supports on professional identity through meaning in life was also significant (Value 0 was not included in the 95% Boot confidence intervals).

**Table 4:** Bootstrap method mediated effect test for the relationship between categorical two-way social support and professional identity

Relative effect type	Effect value	Se / Boot SE	LLCI/ Boot LLCI	ULCI / Boot ULCI
Relative total effects				
X1	0.563	0.059	0.448	0.679
X2	0.716	0.060	0.600	0.833
X3	0.842	0.053	0.738	0.947
Relative direct effects				
X1	0.350	0.056	0.241	0.459
X2	0.445	0.057	0.334	0.557
X3	0.473	0.053	0.368	0.578
Relative indirect effects X→3→5				
X1	0.002	0.005	-0.005	0.013
X2	0.014	0.008	0.001	0.033
X3	0.018	0.009	0.002	0.038
Relative indirect effects X→4→5				
X1	0.206	0.030	0.151	0.268
X2	0.230	0.031	0.172	0.294
X3	0.316	0.036	0.248	0.389
Relative indirect effects X→3→4→5				
X1	0.005	0.007	-0.010	0.019
X2	0.027	0.009	0.011	0.045
X3	0.035	0.008	0.021	0.052

*Note* Reference group: low two-way social support; X1: mainly receiving social support; X2: mainly giving social support; X3: high two-way social support. 3: Achievement motivation; 4: Meaning in life; 5: Professional identity

## Discussion

Based on the two-way social support theory, this study explored the effects of receiving and giving social support on health professional students’ professional identity. Based on the analysis results of Model 1 and Model 2, the study found that both receiving and giving social support had identical roles in enhancing individuals’ positive psychological experiences such as achievement motivation, meaning in life, and professional identity. The current results not only verified Hypothesis 1 we proposed in this study, but also facilitated the practice of the two-way social support theory in relevant fields. From the perspective of medical colleges and medical educators, it is necessary to focus on and promote the ability and level of health professional students to give social support. During the pandemic, health professional students, even when receiving more social support, might experience adverse effects on their mental health [25]. Conversely, health professional students engaged in COVID pandemic prevention and control gained a sense of belonging, and their sense of responsibility and motivation were enhanced [80]. Various community involvements and volunteer services in free clinics or nursing homes (including screening and healthcare for vision, hearing, dental, and basic diseases) serve as interventions to draw insights for promoting health professional students’ ability to provide social support [27, 81, 82].

In this study, we found that achievement motivation had an independent mediating role between receiving social support and professional identity, which was consistent with the results of Huang [49] et al. Based on Herzberg’s Two-Factor Theory, receiving social support could promote some external factors relating to health professional students’ professional satisfaction, such as occupational environment and interpersonal relationships, and thereby enhance achievement motivation [83]. health professional students’ achievement motivation refers to their intrinsic drive to achieve academic and professional achievement. When health professional students feel satisfied and successful, their sense of professional identity also strengthens. We also found that achievement motivation had an independent mediating role between giving social support and professional identity. According to self-determination theory, giving social support could help health professional students construct personal autonomy, a sense of competence, and the need for interpersonal relationships, which was able to promote their self-motivation and motivation formation, and thus enhance professional identity [84]. 

The present results also suggested that meaning in life had an independent mediating effect between receiving social support and professional identity. As was described in social identity theory, receiving social support could help individuals develop a sense of belonging and improve their social identity. Acting as a foundation of meaning in life, social identity and the sense of belonging could promote positive psychological experiences and professional identity [85, 86]. In addition, the theory of self-transcendence proposed by Victor Frankl suggested that individuals could find purpose and meaning in life by transcending their selves, contributing to others, and pursuing higher values [87]. Meaning in life promoted the formation of professional identity [63]. Therefore, the independent mediating role of meaning in life between giving social support and professional identity was supported by the self-transcendence theory. The effect of receiving social support on professional identity through meaning in life was greater than through achievement motivation. As revealed from a conceptual perspective, professional identity was formed under the influence of external factors such as education and clinical environment, which was regulated by the external environment as well as meaning in life [4, 51]. Whereas, achievement motivation was the internal motivation for success in the process of completing tasks, which was also influenced by some external factors, but was more determined by internal factors [40]. Therefore, the influence of social support on professional identity through meaning in life was stronger than that through achievement motivation.

The present findings also suggested that achievement motivation and meaning in life had chain mediation effects between receiving and giving social support and professional identity. Siwek et al. confirmed that achievement motivation was the premise of meaning in life [88]. Intrinsic motivation and identification contributed to an increased desire for exploring, self-awareness, and integration in individuals, making these motivations a possible basis for developing meaning in life [89]. In addition, the present study and previous studies found that achievement motivation had a positive predictive effect on meaning in life [65]. Thus, they were possibly to act as a chain mediator between social support and professional identity. Compared to its independent mediating effect, the chain mediating effect of achievement motivation between receiving and giving social support and professional identity was greater. Therefore, to enhance the influence of achievement motivation on health professional students’ professional identity, promotion of meaning in life while promoting their achievement motivation could be an efficient way. At the same time as the mediating effect is established in the chain of achievement motivation and meaning in life, receiving and giving social support significantly predict professional identity, suggesting that achievement motivation and meaning in life played a partially mediating role in the process. Huang et al. [49] found that achievement motivation and subjective well-being had a chain mediating effect between receiving social support and professional identity, and that subjective well-being played a more important mediating role than achievement motivation. Huang’s results were similar to the findings concerning the role of meaning in life in the chained-mediation in the present study. In addition, studies confirmed that meaning in life and the feeling of well-being were correlated [90, 91]. The present study provided theoretical implications for future investigations on the role of meaning in life and subjective well-being in the relationship between social support and professional identity. The research results supported the effectiveness of interventions in achievement motivation and meaning in life in enhancing professional identity. Medical educators can promote the enhancement of achievement motivation and the perception and pursuit of meaning in life, and thereby facilitate the formation of professional identity by providing learning and training opportunities [92], formulating reasonable assessment mechanisms and reward measures, engaging in positive life education interventions [93], and implementing service-learning (applying academic knowledge to serve society) [94].

Based on the results of Model 1 and Model 2, it was observed that both receiving and giving social support have similar effects in enhancing individuals’ positive psychological experiences (achievement motivation, meaning in life, and professional identity). As indicated by the findings of Model 3, when receiving and giving social support coexist, giving social support can indirectly predict professional identity through achievement motivation and meaning in life. However, receiving social support does not produce the same effects on achievement motivation, meaning in life, and professional identity. Based on the results of Model 1 and numerous scholars [32, 46, 61], this study suggests that this phenomenon may be related to the stronger predictive efficacy of giving social support compared to receiving social support, thereby overshadowing the predictive effects of receiving social support.

Based on the two-way social support theory, this study investigated the predictive efficacy of different types of two-way social support on health professional students’ professional identity. The results suggested that two-way social support could better predict health professional students’ professional identity than one-way social support, taken low two-way social support as control. This effect existed in the total effect, direct effect, independent mediating, and chain mediating effects between two-way social support and professional identity, which verified Hypothesis 5. In addition, we also found that the predictive efficacy of different types of two-way social support on individuals’ positive psychological experiences is hierarchical, which was not mentioned in the theories. The four different types of two-way social support could be ranked according to their predictive efficacy as follows: two-way social support > mainly giving social support > mainly receiving social support. This relationship could also be found in the total effect, direct effect, independent mediator, and chained-mediating effect between two-way social support and health professional students’ professional identity. It indicated that health professional students who mainly gave social support were more likely to maintain positive psychological conditions compared to those who mainly received social support. This discovery aligns with the results obtained from Model 3 in our study. However, the relative mediating effect of achievement motivation on professional identity was not significant in health professional students who mainly received social support, compared to health professional students who received low levels of two-way social support. Whereas the relative mediating effect of meaning in life on professional identity was significant in health professional students who mainly received social support, compared to a low two-way social support group. The results might be attributed to the lower mediating effect of achievement motivation between two-way social support and professional identity, thus leading to a higher threshold of two-way social support. In addition, previous studies showed that receiving social support had positive effects on promoting positive psychological experiences [95, 96]. Based on the above, it could be concluded that two-way social support > primarily giving social support > primarily receiving social support > low level of two-way social support. This study applies the theory of two-way social support to practice, not only validating the practical guiding value of the theory but also proposing new perspectives based on health professional students. Both bidirectional social support theory and community practice theory emphasize the importance of interpersonal interaction and mutual assistance (resource sharing) in professional identity. However, there are some differences between the two: bidirectional social support theory emphasizes the balance of interaction, while community practice theory highlights the role of community involvement. They complement each other, providing theoretical guidance for explaining the formation of professional identity. From a theoretical to practical perspective, the research results suggest to medical educators that merely focusing on the social support received by health professional students is far from sufficient. Educators should pay attention to the role of providing social support, promoting a balanced enhancement of both receiving and providing social support for health professional students. In addition, despite the significant development of online education [97], whether online education is excessive, and whether there is a lack of offline education and community participation in learning and volunteer services, deserves more attention from medical education decision-makers.

### Research limitations

The study did not use stratified random sampling. The representativeness of the sample needed to be improved. The study was a cross-sectional study, which did not demonstrate the relationship between receiving and giving social support and professional identity across a longitudinal span. The measurement tools used in the study may not be entirely suitable for the Chinese university student population. Future research could consider selecting assessment tools with stronger psychometric properties, such as a dedicated scale for providing social support, which might help mitigate the confounding effects of the high correlation between giving and receiving social support. Additionally, there might be self-report biases among some students, where individuals tend to provide responses that align with societal expectations rather than reflecting their true feelings or situations. In addition, whether the results of this study are applicable to health professional students in internship needs further verification, owing to the differences in the main learning tasks and environment between clinical interns and health professional students. The mediating effect of achievement motivation and meaning in life between receiving and giving social support and professional identity was small, and other mediating factors need to be further explored. In this study, the direct and indirect predictive efficacy of different types of two-way social support on professional identity was compared by relative effect values, which failed to test the significance of the differences between every two variables. Other statistical analysis is needed to provide further evidence.

## Conclusion

Compared with previous studies on receiving or perceiving social support and professional identity, this study analyzed the relationship between social support and professional identity from the perspectives that receiving and giving social supports were bidirectional. We confirmed the chain mediation role of achievement motivation and meaning in life between giving and receiving social support and professional identity. The two-way social support theory suggested that two-way social support was more likely to enhance positive psychological characters than giving or receiving social support. Based on this view, we used a multi-categorical variable mediation analysis and found that two-way social support was more likely to enhance positive psychological characters (achievement motivation, meaning in life, and professional identity). The results were consistent in both direct and indirect effects of two-way social support and professional identity. In addition, based on Model 3 and the classification of the relationship between bidirectional social support and professional identity, the study unexpectedly found a hierarchy in the predictive efficacy of different types of two-way support on individuals’ positive psychological experiences, i.e., two-way social support > mainly giving social support > mainly receiving social support > low two-way social support. This finding suggested the importance of promoting the ability to give social support and emphasizing the balance of bidirectional social support. The present results not only provided valuable information on the ways of enhancing health professional students’ professional identity education but also contributed to the further improvement and development of the two-way social support theory.

## Data Availability

The datasets generated and analyzed during the current study are not publicly available because they contain medical student information that they did not consent to have shared publicly at the individual level but aspects of the data set may be available from the corresponding author on reasonable request.

## Abbreviations

COVID-19: Coronavirus disease 2019
PIF: Professional identity formation
PI: Professional identity
AMS: Achievement Motivation Scale
AMS-SF: Short Form of Achievement Motive Scale
Ms: Motive for success
Mf: Motive for avoiding failure
MIL: Meaning in life
