# Correction: The relationship between social support and professional identity of health professional students from a two-way social support theory perspective: chain mediating effects of achievement motivation and meaning in life

**DOI:** 10.1186/s12909-024-05622-9

**Published:** 2024-07-26

**Authors:** Jian Luo, Xiao-Bo Liu, Qian Yao, Yi Qu, Jin Yang, Ke Lin, Shi-Rong Pan, Tian-Yi Wang, Yun Dai, Huan-Yu Chen, Jian-Min Chen, Zheng Yang

**Affiliations:** 1 School of Nursing, Chengdu Medical College, Chengdu, 610500 China; 2 School of Acupuncture and Tuina, Chengdu University of Chinese Medicine, Chengdu, 611137 China; 3 Security Department of Chengdu Medical College, Chengdu, 610500 China; 4 School of Clinical Medicine, Chengdu Medical College, Chengdu, 610500 China; 5 School of Basic Medicine, Chengdu Medical College, Chengdu, 610500 China

**Correction: Luo et al. BMC Medical Education (2024) 24:473**

10.1186/s12909-024-05391-5

Following publication of the original article [1], the authors identified some errors. They would like to add the analysis software (Amose 28.0) to the methods of the abstract (Data were analyzed by use of SPSS26.0 software, Amos 28.0 software, and PROCESSv4.0 plug-in.). In addition, the absence of reference 58 and the duplication of reference 75. Reference 96 needs to be repositioned to reference 58, and reference 97 needs to be moved to reference 77.

Also, the author equal contribution statement has been corrected.

The incorrect equal contribution statement is: Jian Luo^1†^, Xiao-Bo Liu^2†^, Qian Yao^1†^, Huan-Yu Chen^5†^*, Jian-Min Chen^5†^* and Zheng Yang^5*†^.

The correct equal contribution statement is: ^†^ Jian Luo, Xiao-Bo Liu and Qian Yao contributed equally to this work.

The original article has been corrected.

## References

[1] Luo, et al. BMC Med Educ. 2024;24473. 10.1186/s12909-024-05391-5.

